# GLP-1 receptor agonists in Alzheimer’s and Parkinson’s disease: endocrine pathways, clinical evidence, and future directions

**DOI:** 10.3389/fendo.2025.1708565

**Published:** 2025-11-20

**Authors:** Ayush Gandhi, Alireza Parhizgar

**Affiliations:** 1 Department of Hospital Medicine, University of Texas MD Anderson Cancer Center, Houston, TX, United States; 2 Department of Emergency Medicine, University of Texas MD Anderson Cancer Center, Houston, TX, United States

**Keywords:** GLP - 1, Alzheimer, Parkinson, insulin resistance, neurodegenerative disease

## Abstract

Initially developed for type 2 diabetes and obesity, glucagon-like peptide-1 receptor agonists (GLP-1RAs) are now emerging as promising candidates for modifying the course of neurodegenerative diseases. This potential stems from the presence of GLP-1 and its receptors within the central nervous system (CNS), where their signaling activity influences critical processes like synaptic plasticity, neuroinflammation, insulin signaling, and cellular energy management. In fact, preclinical models of both Alzheimer’s disease (AD) and Parkinson’s disease (PD) have shown that GLP-1RAs can reduce neuroinflammation, improve mitochondrial function, and enhance the clearance of toxic proteins (proteostasis), leading to benefits in cognition and the survival of dopaminergic neurons. Yet, when tested in humans, the picture has been more nuanced and less straightforward. Early clinical trials in AD have produced mixed cognitive signals, though they have shown intriguing biological effects, such as preserved cerebral glucose metabolism with liraglutide on FDG-PET scans. In contrast, the evidence in PD has been more consistent, with agents like exenatide and lixisenatide demonstrating motor benefits, although one trial with a pegylated exendin (NLY01) did not meet its primary endpoint. The definitive test will come from large, ongoing phase 3 programs, such as the EVOKE and EVOKE+ trials for semaglutide. Should these trials are successful, GLP-1RAs could become a cornerstone of earlier, mechanism-based intervention strategies for neurodegenerative diseases.

## Introduction

Alzheimer’s disease (AD) and Parkinson’s disease (PD) place an ever increasing burden on public health, driving both disability and rising healthcare costs. Our understanding of these diseases has evolved beyond a simple focus on protein aggregation. Although Alzheimer’s and Parkinson’s disease are classically neurological disorders, their biology intersects strongly with endocrinology through insulin resistance, metabolic failure, and neuroinflammation. This overlap justifies a cross-disciplinary lens and situates GLP-1 agonists within the endocrinology domain (1, 2).

This overlap between metabolic dysfunction and neurodegeneration explains the growing excitement around GLP-1RAs. While systemically known for improving blood sugar, reducing weight, and providing cardio-renal benefits, these agents also activate key pathways within the CNS (like cAMP/PKA, PI3K/AKT, and AMPK). These pathways are known to enhance mitochondrial function, reduce inflammatory signaling through NF-κB/NLRP3, and promote cellular cleanup via autophagy (1, 2).

## GLP-1 and GLP-1R in the CNS: distribution and access

While traditionally known as a gut hormone produced by L-cells, GLP-1 is also synthesized directly within the brainstem by preproglucagon neurons. These neurons project to vital areas including the hypothalamic, limbic, and cortical regions. Complementing this, GLP-1 receptors are found on both neurons and glial cells in the hippocampus, cortex, hypothalamus, and basal ganglia, establishing a clear anatomical network for GLP-1 signaling within the brain (1, 7).

The interaction with the blood–brain barrier partly explains variability across GLP-1 analogues. Smaller molecules or those with less albumin binding may achieve greater penetration, whereas large, pegylated compounds often show limited CNS access. This pharmacokinetic diversity could account for differences in clinical signals across trials (8, 9).

This built-in system suggests the potential for GLP-1 receptor agonists such as exenatide, liraglutide, and semaglutide to influence central pathways. However, whether these drugs achieve meaningful brain concentrations in humans remains debated. Some studies report measurable CNS penetration, while others find minimal or no direct entry, concluding that much of the effect may be mediated indirectly through peripheral or vagal mechanisms (10). Thus, although brain access is possible, the extent to which it explains clinical signals remains an open question. Furthermore, activation of GLP-1 receptors may also help restore the integrity of the blood-brain barrier, a mechanism of particular relevance in the context of aging and neurodegeneration (1, 2). However, how much of these drugs actually reaches the brain is a complex issue, varying based on a drug’s molecular size, its tendency to bind to albumin, and the administered dose. It remains an important open question whether designing drugs for greater brain penetration would actually improve clinical outcomes or simply introduce new toxicities (1, 2).

## Mechanistic convergence: Why GLP-1RAs are biologically plausible in AD/PD

GLP-1RAs fit neatly into the biology of AD and PD—their mechanisms overlap with many of the diseases’ core features. Specifically, GLP-1RAs appear to counter neurodegeneration by: enhancing insulin/AKT signaling, which in turn inhibits GSK-3β, an enzyme implicated in tau pathology; boosting mitochondrial biogenesis and the production of cellular energy (ATP); tamping down neuroinflammation by suppressing microglial activation and inflammasome signaling; promoting the cellular cleanup of toxic proteins through autophagy and lysosomal clearance; and providing neurotrophic support by activating pathways like CREB/BDNF that help maintain neuron health (1, 2).

A unifying thread across these mechanisms is brain insulin resistance, sometimes referred to as ‘type 3 diabetes.’ This state is invariably coupled with micro-inflammation, which in the brain promotes neuronal and glial injury and accelerates amyloid plaque accumulation (11).

At the same time, it’s worth recognizing that much of the benefit seen in trials could just as easily be explained by systemic effects better insulin sensitivity, dampened inflammation, and improved vascular health that secondarily ease the burden on the brain, rather than by direct neuronal stimulation alone (12).

## Clinical evidence in Alzheimer’s disease

The rationale for testing GLP-1RAs in Alzheimer’s disease is straightforward: AD brains are characterized by insulin resistance, reduced energy metabolism, inflammation, and a failure to clear toxic proteins, all processes that are favorably modulated by GLP-1RA signaling. Early clinical evidence supports this concept. A 26-week randomized trial found that liraglutide prevented the decline in the brain’s glucose metabolic rate, as measured by FDG-PET scans, when compared to placebo. Although cognition didn’t change over this short period, the PET findings suggest the drug was hitting its biological target—effects that may show up clinically only later (3). Recent ELAD reports note that liraglutide improved temporal lobe and cortical MRI measures compared with placebo, though the primary metabolic endpoint was not met. Conference data also hint at slower gray matter loss and modest cognitive preservation, suggesting biological activity but falling short of clear clinical efficacy (13, 14) (**Table 1**).

**Table 1:** Mechanistic actions of GLP-1RAs relevant to neurodegeneration (Mechanistic pathways are adapted from prior foundational work on GLP-1 biology and neurodegeneration.

Pathway	Primary nodes	Expected CNS consequence
Insulin signaling	IRS-1 → AKT ↓GSK-3β	Improved synaptic plasticity; ↓tau phosphorylation
Bioenergetics	AMPK, PGC-1α/NRF-1/TFAM	↑Mitochondrial function; ↑ATP; stress resilience
Inflammation	↓NF-κB, ↓NLRP3; microglial M1→M2	↓Cytokines; neuroprotection
Proteostasis	↑Autophagy, lysosome	Clearance of Aβ/tau/α-synuclein
Neurotrophic	↑BDNF/NGF; PKA/CREB	Synaptic maintenance; neurogenesis
BBB/vascular	Tight junction support	↓Leak; improved milieu

References are provided to support each mechanistic pathway) (1, 2, 6, 7).

Recent updates in clinical and observational research have broadened the landscape for GLP-1 receptor agonists (GLP-1RAs) in Alzheimer’s disease. Early-phase trials with liraglutide have demonstrated preservation of cerebral glucose metabolism and slower cortical decline, supporting a biologically active central effect (15–17). The ongoing phase III EVOKE and EVOKE+ programs with semaglutide are designed to determine whether these metabolic and imaging benefits translate into measurable cognitive gains (15, 16). EVOKE enrolls patients with early symptomatic AD, while EVOKE+ targets an even earlier prodromal/MCI stage, allowing direct comparison of intervention timing (6, 18). Meanwhile, real-world analyses of patients with diabetes and obesity receiving semaglutide or tirzepatide have reported lower rates of dementia and cerebrovascular events (19, 20), hinting that these therapies may modify neurodegenerative risk through broader metabolic and vascular pathways. Collectively, these findings reinforce the rationale for targeting GLP-1 signaling in early symptomatic and prodromal stages of Alzheimer’s disease. *Post-hoc* analyses from the REWIND trial suggest that diabetic patients receiving dulaglutide had modestly slower decline in cognitive measures compared with placebo, though these findings were exploratory and should be interpreted cautiously (21).

From a safety perspective, GLP-1RAs are generally well-tolerated. However, the primary side effects of nausea and weight loss are a significant consideration, especially in frail or underweight patients with AD. Across AD trials, dropout has often been higher in the active arm because of nausea and weight loss. These side effects mirror what is seen in diabetes and obesity trials, but their impact may be magnified in frail or underweight patients, making supportive strategies essential (1, 2).

## Clinical evidence in Parkinson’s disease

The scientific reasoning for exploring GLP-1RAs in Parkinson’s disease is similarly strong, as PD is characterized by deficits in insulin/IGF-1 signaling, mitochondrial dysfunction, neuroinflammation, and the toxic accumulation of α-synuclein. All pathological domains influenced by GLP-1RA activity. In PD, the human data so far have been especially encouraging. A landmark phase 2 randomized trial showed that once-weekly exenatide improved motor scores (in the ‘off-medication’ state) over 48 weeks, with these benefits impressively persisting even after the drug was stopped (4). This result echoed earlier open-label studies that had already hinted at a lasting effect (22). More recently, a multicenter trial found that lixisenatide reduced the progression of motor disability over 12 months in patients with early PD, although gastrointestinal side effects were more frequent with the drug (23). Not all results have been positive, however; a trial of the pegylated exendin NLY01 failed to show an overall improvement in motor or non-motor symptoms, though some exploratory analyses suggested a potential effect in certain age groups (5). A Cochrane analysis of the available data supports a possible motor benefit but rightly emphasizes that the evidence is of low-to-moderate certainty, highlighting the need for larger and longer studies (24) (**Table 2**).

**Table 2:** Selected clinical trials.

Indication	Agent	Population & duration	Primary outcome	Signal summary
AD (mild)	Liraglutide	n=38; 26 wks	FDG−PET CMRglc	Prevented decline vs placebo; no cognitive separation (3)
AD (mild)	Liraglutide (ELAD)	n≈200; 52 weeks	FDG−PET; cognition	Protocol published; results pending/ongoing analyses (14)
AD (early symptomatic)	Semaglutide (EVOKE/EVOKE+)	n=2000; 104 weeks	Clinical & biomarker endpoints	Phase 3 design published (6)
PD (moderate)	Exenatide QW	n= 62; 48–60 wks + washout	OFF motor score	Improved vs placebo; persists off−drug (4)
PD (early)	Lixisenatide	n=156; 52 weeks + short washout	MDS−UPDRS III	Less motor progression than placebo (23)
PD (early)	NLY01	n= 453; 48 wks	MDS−UPDRS	Primary endpoint not met; age subgroup signal (5)

Recent trials have continued to strengthen the clinical signal for GLP-1 receptor agonists (GLP-1RAs) in Parkinson’s disease. Phase II studies of exenatide and lixisenatide have shown consistent improvements in motor performance, with benefits that occasionally persist after treatment discontinuation (15, 25–27). These results complement mechanistic data suggesting enhanced mitochondrial function, reduced inflammation, and improved dopaminergic neuron survival. Observational cohorts further indicate that individuals with diabetes treated with GLP-1RAs or newer dual agonists such as tirzepatide may have a lower incidence of Parkinson’s disease or slower progression of motor decline (19, 20). Although confirmatory phase III trials are still needed, the overall pattern points to a genuine disease-modifying potential for this drug class.

One reason the PD data appear more convincing is that motor scales, such as the MDS-UPDRS, tend to capture changes over months, whereas cognitive measures in AD progress more slowly and require longer follow-up to show separation. In addition, PD trials have often included washout periods, which provide a cleaner test of whether benefits persist beyond symptomatic effects. Together, these design features may partly explain why signals in PD look stronger than in AD, despite similar underlying biology (28).

As with AD, tolerability in PD patients mirrors what is seen in metabolic diseases. Proactive management of potential weight loss is crucial, particularly for leaner patients, to ensure it does not become problematic. Similarly, in PD cohorts, gastrointestinal side effects like nausea and reduced appetite account for most of the excess discontinuations. The frequency is broadly comparable to what’s reported in metabolic trials, though it becomes clinically more concerning in patients who already struggle to maintain weight.

## Controversies and open questions

A key question is whether the neuroprotective effects are limited to patients with diabetes or obesity. Many of the proposed mechanisms, such as anti-inflammatory and proteostatic benefits, are theoretically independent of blood sugar control; it is possible that patients who respond best may be those with underlying insulin resistance or a high inflammatory burden, phenotypes that future trials should carefully measure. While some benefits may reflect improved glycemic control, mechanistic pathways such as reduced neuroinflammation and enhanced proteostasis are not glucose-dependent. Similar reductions in dementia incidence have been observed after bariatric surgery, again suggesting that improvements in metabolic and inflammatory milieu—rather than direct central actions may be the critical link (29). Future trials must stratify outcomes by diabetes status to clarify whether efficacy is disease-specific or generalizable (1, 2, 20).

Is this a class effect? The evidence so far suggests that GLP-1RAs have more potent neurological signals than several other classes of glucose-lowering drugs. Whether newer dual-agonists that target multiple receptors will offer even greater benefits is an active area of investigation. Of note, other metabolic drugs such as metformin and SGLT-2 inhibitors also show preclinical neuroprotective effects, though clinical data remain sparse compared to GLP-1RAs (30–32).

Another open question is timing. When to start treatment and how long to continue it? It seems likely that earlier intervention at the stage of MCI (Mild cognitive impairment) or early PD and longer treatment durations of at least 18 to 24 months will be critical for success. The therapeutic window for GLP-1RAs is probably limited to the very early stages of disease, when neurons and glial cells are still viable. Once these cells have been lost, there is little left to preserve, making early recognition and timely intervention critical. The signals of durable effects after washout in PD trials are encouraging but are not yet definitive proof of true disease modification (4, 5, 23, 24).

Finally, there is the issue of drug delivery. While designing agents that cross the blood–brain barrier more effectively seems attractive, greater brain penetration usually comes with higher systemic exposure. That trade-off could amplify side effects like nausea, GI upset, or unwanted weight loss issues that are especially difficult to manage in frail or elderly patients (1, 2, 8).

## Practical implications for endocrinologists

At present, GLP-1RAs are not indicated for Alzheimer’s or Parkinson’s disease. Endocrinologists should limit their use to approved metabolic indications. Neurologists, likewise, lack sufficient evidence to prescribe GLP-1RAs specifically for neurodegeneration outside trials. For practicing endocrinologists, the current evidence calls for a balanced approach. The primary reason to prescribe GLP-1RAs should remain their approved metabolic indications, and we must be careful not to overstate the potential neurological benefits to patients outside of a clinical trial setting. That said, for patients with T2DM or obesity who are also at high risk for neurodegeneration, choosing a GLP-1RA might offer the potential for ancillary neuroprotection, though we must await definitive phase 3 outcomes before making such claims (4–6, 22, 23). Importantly, initiating these therapies requires a multidisciplinary mindset, anticipating common side effects like weight loss and GI issues and proactively managing them with nutritional support and recommendations for resistance exercise.

Extending the use of GLP-1 receptor agonists (GLP-1RAs) to older and frail individuals requires thoughtful clinical judgment. The most frequent side effects are gastrointestinal, such as nausea or vomiting, which can reduce appetite and lead to unintended weight loss, an outcome that can be harmful in patients who are already underweight or vulnerable (33–35). These agents may also contribute to dehydration, gallbladder complications, and, in rare cases, pancreatitis. Current guidance from the American College of Cardiology and the American Diabetes Association emphasizes slow dose escalation, routine monitoring of body weight, and the incorporation of nutritional support, particularly in older adults (33–35). In frail elderly patients, even moderate weight loss can worsen muscle mass and functional ability unless balanced by adequate protein intake and resistance exercise. For this reason, patient selection and ongoing monitoring should be deliberate, with attention to maintaining strength and nutritional stability throughout treatment.

## Conclusion

GLP-1RAs offer a therapeutic strategy that targets the intertwined metabolic and inflammatory networks increasingly recognized as central to neurodegeneration. To date, the clinical evidence is strongest for motor benefits in Parkinson’s disease, with biologically plausible but less clinically advanced data in Alzheimer’s disease. The next step is longer, larger trials that start early in the disease course. These future studies must also be smarter, stratifying patients by their metabolic and inflammatory profiles and using rigorous, mechanism-based endpoints like measures of insulin signaling, autophagic flux, or the actual burden of tau or α-synuclein in the brain (6). If these upcoming trials confirm the efficacy of GLP-1RAs, it could herald a new, endocrinology-informed era of treating neurodegenerative diseases, one where we use multimodal strategies that combine metabolic reprogramming with other targeted agents to preserve function and truly delay disability.
